# Service Dogs and Persons with Disabilities: When COVID-19 Lockdown Changes Their Relationship

**DOI:** 10.3390/ani13050914

**Published:** 2023-03-02

**Authors:** Marine Grandgeorge, Céline Rochais, Florian Auffret, Nicolas Dollion

**Affiliations:** 1 EthoS (Éthologie Animale et Humaine)-UMR 6552, CNRS, University Rennes, Normandie University, 35380 Paimpont, France; 2 Association Handi’Chiens, 43-45 Rue Pierre Valette, 92240 Malakoff, France

**Keywords:** COVID-19 lockdown, interspecific relationship, service dog, social isolation

## Abstract

**Simple Summary:**

COVID-19 constitutes a major event with multiple consequences for our intra- and interspecific relationships. Persons with disabilities, who own service dogs, develop strong relationships with them. In the present study, we hypothesized that the COVID-19 lockdown would have influenced people with disabilities/service dog relationships. An online survey was conducted during the first COVID-19 lockdown in France, which included information on the general context prior to and during the COVID-19 lockdown. The results confirm that service dogs, like other pets, constituted a source of emotional support for people during the COVID-19 lockdown. However, it appears that the COVID-19 lockdown elicited a costlier relationship for service dog owners. Our study highlights that, in extreme situations, the characteristics of human–animal relationships can be exacerbated in positive and negative ways. These results are important for organizations providing service dogs to evaluate the support they provide to their recipients.

**Abstract:**

Persons with disabilities, who own service dogs, develop strong relationships with them. Since the COVID-19 pandemic decreased the possibility of social contact and modified human relationships, we hypothesized that the COVID-19 lockdown would influence people with disabilities—service dog relationships. An online survey was conducted during the first COVID-19 lockdown in France and included information (e.g., MONASH score) both in the general context prior to and during the COVID-19 lockdown. Seventy owners participated. Compared to the general context, scores for the Perceived Emotional Closeness and Perceived Costs subscales were significantly higher during the COVID-19 lockdown, while scores for the Dog–Owner Interaction subscale were significantly lower during the COVID-19 lockdown. Our study confirmed that service dogs, like other pets, were a source of emotional support for their owners during the COVID-19 lockdown. However, people with disabilities found their relationship with their service dog costlier (e.g., my dog makes too much mess). Our study highlights that, in extreme situations, characteristics of a human–animal relationship can be exacerbated in both positive and negative ways.

## 1. Introduction

According to Hinde [1], “a relationship between two individuals involves a series of interactions […] over time, such that each interaction may be affected by preceding ones”. Relationships are thus not a fixed entity but are dynamic over time since various emotional processes are involved—such as feelings, hopes and distress—which may influence individual expectations of the relationship. Furthermore, this bond is built between two individuals, and the cultural, temporal and social contexts in which a relationship is embedded must be considered since these factors may influence the relationship [1]. For example, stress or other challenges in families (e.g., unemployment) can result in a reduction in satisfaction in a relationship [2,3].

Although relationships and interactions were historically conceptualized in intraspecific contexts, these concepts can be applied at the interspecific level [4]. Thus, like the relationships and interactions between humans, external factors can influence human–animal relationships and interactions. For example, inappropriate human management of horses (e.g., food, spatial and social restriction) may compromise the horse’s welfare and consequently alter the human–horse relationship [4,5,6]. Additionally, just as peers and family represent an essential part of the human environment, pets are often partners in our immediate environment [7]. Families, including animals, could thus be viewed as a group with ongoing and constantly changing interactions among members [8]. As a consequence, similar to human intraspecific relationships [1], interspecific relationships with pets may be impacted by major contextual changes.

One could argue that the worldwide health context associated with COVID-19 is a major event with multiple consequences on our intra- and interspecific relationships. In less than three months, at the end of January 2020, a public health emergency was declared by the World Health Organization, and 6 weeks later, it was upgraded to pandemic status, i.e., the COVID-19 pandemic [9]. Consequently, unprecedented restrictions on movements, travel, work, social contacts and leisure activities were set globally to prevent the spread of the virus. For example, in France, the first lockdown was enforced for 2 months from 17 March 2020 (with an additional month with strict movement restrictions). Additionally, the COVID-19 pandemic clearly caused stress and anxiety, affected mood, and sometimes, in extreme situations, increased the risk of developing post-traumatic stress disorders and suicides, especially due to social isolation (e.g., [10,11,12,13,14]). 

Different studies demonstrated that, in this specific context, pets and especially dogs alleviated the negative impacts of the COVID-19 lockdown in the general population. For example, in the USA, using an online survey during the first month of COVID-19 (April 2020), Bussolari et al. [15] investigated the thoughts, experiences and concerns of adults living with pet dogs. Having a pet dog clearly reduces the feeling of isolation and loneliness, as well as sustains both the mental and physical health of their owners. Similar benefits were observed in British pet dog owners between April and June 2020 [16,17] and Spanish owners in March 2020 [18]. Furthermore, pet cats were valuable and comforting human companions during lockdowns, as were other animals [17,18], but to a lesser extent [17]. 

As expected, the COVID-19 pandemic, including lockdown periods, influenced the owners’ interspecific relationship with their pets. For example, using a questionnaire based on the Monash Dog–Owner Relationship Scale or MDORS [19] to evaluate the social support obtained from pet dogs, Bowen et al. [18] showed that, during the lockdown, pet dog–owner relationships differed from that in their normal daily life context. Owners engaged in more shared activities with their dogs, hugged them more often, turned to them more as a source of companionship and comfort, and confided more in them. However, using a revised version of MDORS, Howell et al. [20] reported that although dogs can be social substitutes for humans in some crisis situations involving social isolation, such as COVID-19 lockdowns, their influence was not as great as expected, since humans could keep in touch with one another via telecommunications [21]. Consulting over 400 diaries, Holland et al. [22] found specific changes in pet dog–owner relationships during the lockdown, e.g., pet dogs enjoying increased human company or more time for their training.

However, as explained by Bowen et al. [18] and Christley et al. [23], pets can also experience the negative consequences of lockdown since their quality of life “is highly influenced by the characteristics of their physical and social environment, as well as the behaviour and lifestyle of their owners [24], all of which would be substantially changed during an official lockdown” [18]. Consequently, pet dogs displayed more behavioral problems (e.g., object destruction, barking all day) during lockdown periods, probably resulting from changes in their daily routines. For example, around 80.0% of UK dog owners reported that their dog’s routine had changed compared to pre-lockdown [23]. Most Spanish dog owners reported that their dog’s behaviors “got worse”, with more attention-seeking behavior (41.6%), annoying or excessive vocalizations (24.7%) or excitable behavior (20.8%) [18]. In another British survey, pet dog owners reported, in their diaries, new undesirable behaviors during the lockdown period, such as attention-seeking or destructive behaviors [22]. One could argue that these changes are subject to variation according to the populations surveyed and the type of questions asked. For example, only 3.2% of English-speaking respondents (e.g., USA, UK, Canada, Australia) mentioned a strained relationship with their pet dog [15]. In addition, results could be different if the animals at home are not pets but trained animals such as service dogs. 

Service dogs have been specifically trained to cope with particular situations and to perform a specific physical or functional task(s) to aid child- adolescent- and adult-owners with various forms of disabilities [25]. First used to help adults with mobility challenges, service dogs are now also trained to help individuals with a wide range of developmental disorders, disabilities or chronic health conditions such as diabetes, post-traumatic stress disorder (PTSD), epilepsy and autism spectrum disorder (ASD) [26]. This training may lead to particular relationships between the owners and their service dogs. For example, using the MDORS, two recent studies indicate that the dog–owner bond—and hence attachment between dog and owner—was significantly higher with seizure-alert dogs than with non-alert dogs [27,28]. Service dogs also provide companionship, e.g., for people with ASD [29,30]. These bonds also lead to numerous benefits for the recipients, as reviewed by Lindsay and Thiyagarajah [25]: quality of life, reduced stress and anxiety, enhanced self-confidence, social interaction and physical health.

Considering the stress, anxiety and loneliness generated by the COVID-19 pandemic, especially during the first lockdown, in various countries around the world [10,11,12,13,14], recipients of service dogs may have also been affected by such difficulties and/or be impacted at a higher (or at least different) level than the general population, as observed in other at-risk populations [31]. Surprisingly, even though Ratschen et al. [17] mentioned that their sample involved 57 service dogs (e.g., guide dogs), no analyses focusing on human–service dog relationships have been done. To our knowledge, no other study mentioned service dog–recipient relationships during the COVID-19 pandemic, especially during the first lockdown period.

There is no doubt that pets, especially pet dogs, provided social support to their owners during COVID-19 lockdowns and that human–pet relationships could change during this period. Thus, we hypothesized that a similar phenomenon would also be observed for humans with disabilities and service dog dyads. Based on the previous literature, we hypothesized that relationships between people with disabilities and their service dogs would become stronger during COVID-19 lockdowns than before. It has been established that recipients have strong relationships with their service dogs in their daily life [25], and COVID-19 lockdowns could change these dyad interactions—and hence, relationships. We investigated the relationship between owners with disabilities and their service dogs in a French population, using an auto-evaluated MDORS questionnaire and three additional sub-scales, as well as measures of other components of daily life prior to and during lockdown (e.g., location, working or not, living alone or not).

## 2. Materials and Methods

### 2.1. Context of the Study

In France, the first cases of COVID-19 were diagnosed at the end of 2019, and a pandemic expansion at the beginning of 2020. The French government established a lockdown of the whole population from 17 March with strict restrictions on time spent outdoors (e.g., 1 h per day for walking within an authorized radius of 1 km from residence). Each family was confined to their own home, without outside visitors, except in case of an emergency. Confinement was progressively reduced with authorization to travel freely within French borders at the beginning of June 2020. We solicited participants and collected data between 15 May and 30 June 2020 (https://covidchienassistance.limequery.com/636695?lang=fr; survey’s link is not available anymore).

### 2.2. Subjects

#### 2.2.1. Ethics

The survey complied with the French law on digital information and was approved by the University of Rennes 1. Participants were fully informed about the aim of the study before the survey completion and informed about the hypothesis of the study after the survey completion. As the study survey was entirely anonymous, informed signed consent was not required from participants. Participants could withdraw from the online survey at any time.

#### 2.2.2. Human Participants

**Figure 1:**
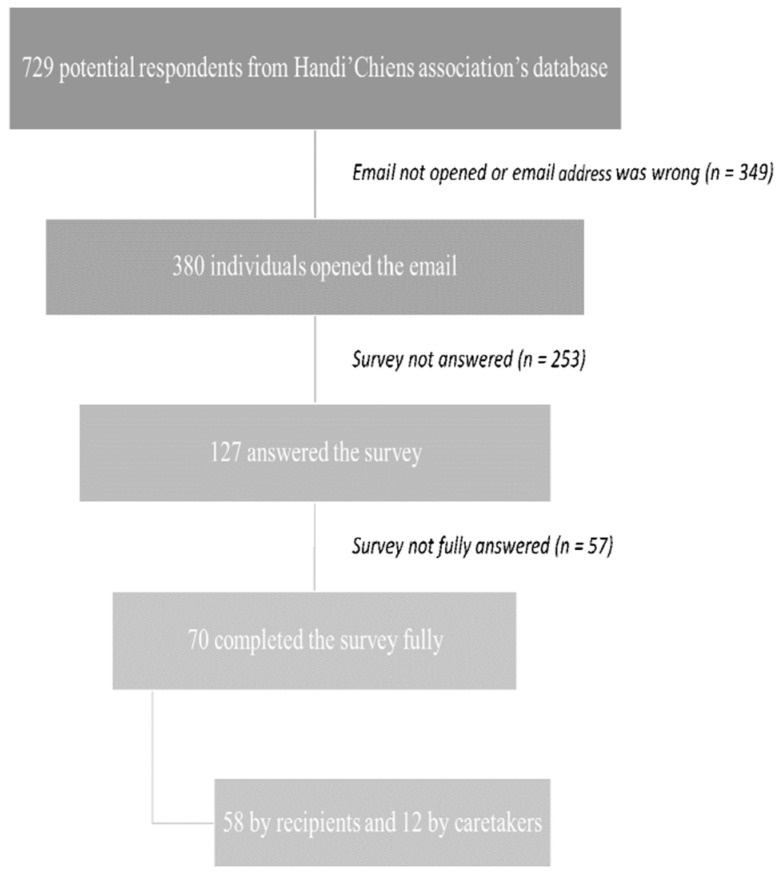
Flow chart showing the survey process and criteria used to include survey’s answers.

Using the Handi’Chiens association’s database (containing contacts of people with disabilities and service dogs), all listed recipients and their families were invited to participate in the survey. The survey was available online on LimeSurvey (i.e., an online surveying tool). We used the contact e-mail addresses in the database to email invitations to 729 potential respondents, providing detailed information about the aim of the survey, as well as the digital link to the online questionnaire. A total of 380 individuals opened the email, and 127 answered the survey (final rate of 17.4%). Among the 127 respondents, 70 completed the survey completely (58 by recipients and 12 by caretakers). A flow chart of the survey process is presented in Figure 1.

### 2.3. Survey Design

The online survey could be completed either by the recipients themselves or with the help of their caretakers (e.g., a recipient’s parent for children with ASD). Data were collected through close-ended questions (e.g., yes/no, male/female), open-ended questions (i.e., comment on the answer when “other” was selected) and a 5-point Likert scale. The survey was performed only at one time during Spring of 2020, and we collected information during the COVID-19 lockdown period and for the general context (i.e., prior to COVID-19 lockdown). “In general context” was explained at the beginning of the survey as corresponding to the condition before and outside COVID-19 lockdown, as the daily life routines.

The online survey included five categories of questions:

1. Recipients’ relationship with their service dog (*n* = 37 questions). This category is composed of two subparts. For the first subpart, we used the Monash Dog–Owner Relationship Scale (MDORS), a validated questionnaire [19] aimed at evaluating human–dog relationships. The MDORS includes 28 questions. The item “How often do you take your dog in the car?” was removed since most recipients did not have a car or drive a car. Thus, our survey was comprised of 27 questions, contributing to three different subscales: (1) Dog–Owner Interaction; (2) Perceived Emotional Closeness; and (3) Perceived Costs. The Dog–Owner Interaction sub-scale corresponds to activities related to the care of the dog and close activities (e.g., kiss, pet, groom the dog). As mentioned previously [19], “such activities indicate the amount of time spent together in a relationship as well as the opportunity for shared emotional experiences and reciprocal interactions, which are known to be important elements in the formation of affectional bonds”. The Perceived Emotional Closeness reflects social support, attachment and companionship, an important component of human–dog bond. The Perceived Costs explore the potential perceived costs associated with a dog by the owner, independent of the perceived benefits [19], such as money, mess and difficulties in looking after the dog. The answers to subscales 1 and 2 were rated from 1 to 5 (never/totally disagree to always/strongly agree), and the answers to subscale 3 were rated from 1 to 5 (never/totally disagree to always/strongly agree). The MDORS was back-translated to French, as previously done [27]. For the present study, each item of the MDORS was adapted to two versions of the questions: a) relationship with the service dog during the COVID-19 lockdown; and b) relationship with the service dog in a general context prior to the COVID-19 lockdown. For example, for item 1 of Perceived Emotional Closeness subscale, people answered the sentence: during general context, my dog helps me get through tough times; then, during this COVID-19 context, my dog helps me get through tough times. Next, people answered item 2 and so on. In the second subpart, additional elements previously pointed out in the literature relative to service dogs were collected, such as the dog’s walks (i.e., frequency and duration) and possible changes in behaviors during the COVID-19 lockdown (i.e., boredom, excitement, objects’ destruction, vocal noises, attention seeking, other).
2. Other animals at recipients’ home (*n* = 1 question). Information was gathered about other dog(s) and/or other species at recipient’s home.
3. COVID-19 contagion context for the recipients (*n* = 6 questions). Participants were asked if and how COVID-19 had impacted them (i.e., recipients), their family and/or their friends. They were asked (1) if they knew anybody personally who had contracted COVID-19, if these people had been (2) hospitalized or (3) died, (4) whether they had been positively-tested for COVID-19, (5) felt COVID-19 symptoms, and lastly (6) to describe their general change in stress level since the beginning of the COVID-19 pandemic (4-point Likert-scale from more stress to no stress). Since these questions could be emotionally difficult, participants could skip them.
4. Recipients’ daily life (*n* = 7 questions). We collected information about the dog recipients’ work or institution, their accommodation generally, and about the impact of COVID-19 on these elements. We asked (1) what kind of activity the recipient had done (e.g., work, school, unemployment), (2) if and how it changed during lockdown (e.g., teleworking), (3) their type of housing during lockdown: lived in their regular residence or had to move, the (4) type of residence: flat, house or other, (5) its location: urban, semi-urban or in a rural area, (6) presence—or not—of an outside area (e.g., garden, balcony), and (7) if they lived alone or not.
5. General information (*n* = 10 questions). Questions from this category yielded general information concerning the recipients (e.g., sex, age) and their service dog (e.g., age, service type, breed). Since France was divided into green zones (i.e., where COVID-19 occurred to a lesser extent, with fewer people at hospitals and fewer deaths due to COVID-19) and red zones (i.e., where COVID-19 was more prevalent, with more people in hospitals and more deaths due to COVID-19) during lockdown, the participants’ regional location was recorded since it may have affected recipients’ perceived stress.

### 2.4. Data Collection and Analyses

Only fully completed questionnaires were considered for analysis (*n* = 70). All results were analyzed using Statistica 13 software (http://www.statsoft.com/textbook/, accessed on 1 July 2022) statistical tests. Since the data did not fit a normal distribution, non-parametric statistics were used [32]. The significance threshold was set at *p* = 0.05. 

Previously, some authors using the MDORS analyzed total scores for all items [33] while others analyzed either mean scores only [34] or both [27]; we chose the last option in this study for 2 reasons: there was no defined procedure, plus these two ways of analyses have previously been shown to complement each other [27]. MDORS scores were calculated for the whole scale and its three subscales (i.e., Dog–Owner Interaction, Perceived Emotional Closeness, and Perceived Costs). First, a total score was calculated by adding the scores of all items (for the whole MDORS and each of its subscales). Then, mean scores (i.e., the total divided by the number of items) were calculated for the three subscales and the total MDORS. These scores on the MDORS were calculated for answers concerning the COVID-19 lockdown period and for answers concerning the period outside COVID-19. The MDORS scores (i.e., total scores and scores for each subscale) and specific items of the three subscales were compared between COVID-19 lockdown and the period outside COVID-19 using Wilcoxon matched pairs test. Spearman correlation tests were used to analyze the relationships between subscales during and prior to COVID-19 lockdown. Spearman correlation tests were used to compare MDORS scores on subscales between contexts (i.e., scores during COVID-19 lockdown *minus* scores for the general context) to evaluate links between variations on subscales. Finally, using Kruskal–Wallis, Mann–Whitney U and Pearson correlation tests, we compared scores (i.e., total and for each subscale) for the MDORS subscales and other parts of the questionnaire (i.e., other animals at home, COVID-19 context, recipient daily life, general information).

## 3. Results

### 3.1. Information about the Participants and Service Dogs

Details about the recipients and their service dogs are presented in Table 1. Overall, 70 recipients answered the survey (*n* = 47 ♀, 67.1%; *n* = 23 ♂, 32.9%; mean age ± SD = 37.4 ± 15.3 years old, range 8 to 72; 10 under 18 years old). The survey was mostly completed by the recipients themselves (*n* = 58), while 12 completed it together with their caretaker (*n* = 11 mother, *n* = 1 father). The highest level of education (for the recipient or for a parent if the recipient was under 18 years old) was a high school diploma (54.2%). The employment status of the recipient, or the highest one for parents if the recipient was under 18 years old, was mainly unemployed (54.2%). Thus, by the time of the survey completion, only a few recipients were employed (22.9%) or went to school or a specialized institution (15.7%). These activities suddenly stopped or changed drastically during lockdown for almost all the participants; only 1 of the 16 employed recipients continued to go to work, while 10 were working online, and all schools/institutions were closed. 

During the COVID-19 lockdown, most recipients did not live alone (71.4%) and were confined in their regular housing (92.9%). Their housing conditions were mainly in a house (64.3%), with an outside area (i.e., at least a balcony: 93.2%) with a quasi-equal distribution between urban, semi-urban and rural areas (35.7%, 28.6%, and 35.7%, respectively). Most lived in an area with low COVID-19 contagion rates (i.e., green area: 65.7%).

The 70 service dogs involved in the survey were well balanced according to sex ratio (*n* = 33, 47.1% ♀; *n* = 37, 52.9% ♂), breed (*n* = 35, 50.0% Labradors; *n* = 34, 48.6% Golden retrievers) and well distributed according to age (mean age ± SD = 5.72 ± 2.48 years old, range 2 to 12). They were mainly service dogs for individuals with physical disabilities (*n* = 62, 88.6%), while others were service dogs for ASD children (*n* = 7, 10.0%) and one was a seizure alert service dog (*n* = 1, 1.4%). Such an unbalanced sample did not allow comparison between types of recipients. All had lived with the recipient for several years (mean ± SD = 42.0 ± 30.7 months). Most recipients (58.6%) owned one or more other animals (i.e., pet dog, cat).

Approximately half of the service dogs were reported by their recipient to have displayed behavioral changes during lockdown (48.7%; Table 1). The most frequently reported changes in these 34 service dog behaviors were human attention seeking (64.7%), boredom (41.2%), vocal emissions (32.3%), excitement (14.7%) and destruction of objects (5.9%) occasionally. Some of the 18 recipients (25.7%) who reported other behavioral changes during lockdown mentioned, for example, a decrease in obedience (in general or specific contexts such as running away; *n* = 3), an increase in time spent sleeping (*n* = 1) and that the service dog became cuddlier and closer (*n* = 4). None mentioned aggressiveness. Interestingly, behavioral changes were not associated with changes in living place (only one recipient who was not living in her regular housing during lockdown reported that her service dog manifested behavioral changes). Similarly, they were not associated with housing conditions during the COVID-19 lockdown (house versus flat: X^2^ = 2.02, *p* = 0.154; urban, semi-urban versus rural: all *p* > 0.05; presence versus absence of an outside area: X² = 0.2, *p* = 0.653; housing alone versus not: X^2^ = 0.02, *p* = 0.879).

### 3.2. Impact of the COVID-19 Context on Relationships between Recipients and Their Service Dogs

For the sake of clarity, only significant results are discussed.

The COVID-19 lockdown modified the relationships between recipients and their service dogs. Scores for the three MDORS subscales differed significantly between the COVID-19 lockdown and the general context prior to COVID-19 lockdown (all *p* < 0.05; Table 2). The Perceived Emotional Closeness subscale scores were significantly higher during the COVID-19 lockdown than for the general context (total score, Z = 1.99, *p* = 0.047; mean score, Z = 2.07, *p* = 0.038). Interestingly, the Perceived Costs subscale scores were also significantly higher during the COVID-19 lockdown than for the general context (total score, Z = 2.17, *p* = 0.030; mean score, Z = 2.10, *p* = 0.036). Conversely, the Dog–Owner Interaction subscale scores were significantly lower during the COVID-19 lockdown than for the general context (total score, Z = 3.30, *p* < 0.001; mean score, Z = 3.30, *p* < 0.001). No significant differences were reported between COVID-19 lockdown (Sum ± SD = 90.46 ± 8.64, Mean ± SD = 3.35 ± 0.32) and general context (Sum ± SD = 90.46 ± 8.7, Mean ± SD = 3.35 ± 0.32) for total scores on the MDORS (Wilcoxon tests, Z = 0.461, *p* = 0.645 and Z = 0.409, *p* = 0.683, respectively).

Comparisons of scores during and outside the COVID-19 lockdown for each item of the MDORS revealed significant differences between contexts for specific items of the three subscales (Table 2). The Perceived Emotional Closeness subscale yielded higher recipient scores during the COVID-19 lockdown than for the general context concerning the trauma the service dog’s death might cause them (Z = 2.02, *p* = 0.043) and the tendency to tell things to their service dog they would not tell anyone else (Z = 2.82, *p* = 0.005). Scores for two items from the Perceived Costs subscale were higher: the difficulty to look after the service dog (Z = 2.76, *p* = 0.006) and the fact that the service dog made too much mess (Z = 2.92, *p* = 0.004) was higher during the COVID-19 context than for the general context. Multiple significant differences were observed for items on the Dog–Owner Interaction subscale: recipients gave significantly more hugs (Z = 3.18, *p* = 0.002) and kisses (Z = 2.52, *p* = 0.012) to their service dog during the COVID-19 lockdown than in the general context; they played less with the service dog (Z = 3.24, *p* = 0.001), bought it fewer presents (Z = 3.76, *p* < 0.001) and took it less with them to visit people (Z = 5.11, *p* < 0.001) during the COVID-19 lockdown than in the general context.

Correlation analysis revealed similar significant relationships between MDORS scores on subscales for the general context and the COVID-19 lockdown. MDORS scores on the Perceived Emotional Closeness subscale were positively correlated with scores on the Dog–Owner Interaction subscale (General context: r = 0.577, *p* < 0.001; COVID-19 lockdown: r = 0.503, *p* < 0.001) and negatively correlated with scores on the Perceived Costs subscale (General context: r= −0.465, *p* < 0.001; COVID-19 lockdown: r = −0.390, *p* < 0.001). Although the correlation was not significant, scores on the Dog–Owner Interaction and the Perceived Costs subscales tended to correlate negatively for the General context only (r = −0.224, *p* = 0.06).

Analyses of differences in MDORS scores on subscales between contexts (i.e., scores for the COVID-19 lockdown *minus* scores for the general context) revealed the presence of a significant positive correlation between score differences on the Perceived Emotional closeness subscale and the Dog–Owner Interaction subscale (r = 0.325, *p* = 0.005). Thus, an increase in the score on the Perceived Emotional Closeness subscale during the COVID-19 lockdown compared to the general context was also associated with an increase in the score on the Dog–Owner Interaction subscale. No other significant correlations on score differences on MDORS subscales were observed.

### 3.3. Factors Affecting the Impact of the COVID-19 Lockdown on Recipient–Service Dog Relationships

Significant differences were found when comparing MDORS scores on subscales (i.e., score during the COVID-19 lockdown *minus* the score for the general context prior to the COVID-19 lockdown) according to recipients’ daily routine and other general information.

First, comparisons revealed that recipients’ scores increased more on the Perceived Costs subscale when they indicated that their service dog manifested: (1) changes in their behavior during the COVID-19 lockdown (*Yes* Mean ± SD = 1.68 ± 3.16, *No* Mean ± SD = −0.33 ± 1.79, U = 329, *p* < 0.001), (2) frequent boredom (*Yes* = 2.43 ± 2.47, *No* = 0.20 ± 2.62, U = 177, *p* = 0.001), (3) increased excitement (*Yes* = 4.40 ± 2.51, *No* = 0.35 ± 2.53, U = 27, *p* < 0.001), (4) more frequent vocal manifestations (*Yes* = 3.64 ± 3.47, *No* = 0.09 ± 2.18, U = 103, *p* < 0.001). The same was true when the participants said they felt more stressed during the COVID-19 lockdown (*Not/Less Stressed* = −0.54 ± 0.53, *Same* = 1.60 ± 0.68, *More Stressed* = −1.10 ± 0.47; H = 11.67, *p* = 0.009; *Not/Less* versus *Same*, *p* = 0.009; *Not/Less* versus *More*, *p* = 0.075) and when their housing did not include a garden (*Yes* = 0.11 ± 2.83, *No* = 1.54 ± 2.54, U = 401, *p* = 0.03). Finally, the scores on the Perceived Costs subscale of participants who indicated that they had shorter walks during the COVID-19 lockdown increased (*Shorter* = −1.80 ± 0.49, *Same* = −0.67 ± 0.73, *Longer* = −0.50 ± 0.89; H = 11.04, *p* = 0.004; *Same* versus *Shorter*, *p* = 0.01).

Scores on the Dog–Interaction subscale of participants decreased more between the general context and the COVID-19 lockdown when they reported that (1) they decreased the frequency of walks with their dog during the COVID-19 lockdown (*No walks* = −3.63 ± 1.01, *Less* = −2.35 ± 0.60, *Same* = −0.33 ± 0.55, *More* = 0.17 ± 0.83; H = 14.07, *p* = 0.003; *Less* versus *Same* and *More*, both *p* < 0.05), (2) they did not remain in their regular housing during this period (*Yes* = −1.0 ± 2.69, *No* = −5.0 ± 5.52, U = 78.5, *p* = 0.05), and (3) their housing did not include an outdoor space (*Yes* = −0.90 ± 2.84, *No* = −3.89 ± 4.20, U = 153, *p* = 0.03). In addition, a significant negative correlation was found with the dogs’ age: younger dogs were associated with a greater increase in the score on the Dog–Interaction subscale (COVID-19 lockdown, r = −0.240, *p* = 0.045).

Finally, we found only one significant difference between the Perceived Emotional Closeness data. Scores of participants who indicated that they had more than one dog (i.e., in addition to their service dog) increased between the general context and the COVID-19 lockdown (*Yes* = 2.30 ± 0.75, *No* = 0.37 ± 0.30, U = 156, *p* = 0.011). However, having other types of animals did not seem to have any significant effects.

## 4. Discussion

Previous studies showed that pets and service dogs provide social support for people and help them cope with difficult situations in their daily life [35,36] and in a crisis such as the COVID-19 pandemic [17,18]. Using the MDORS scale, the present study confirms that service dogs are a source of support for their recipients and demonstrates how the COVID-19 lockdown modified relationships between recipients and their service dogs. While recipients of service dogs perceived more emotional closeness (Perceived Emotional Closeness subscale), they also reported an increase in costs (Perceived Costs subscale) and a decrease in interactions (Dog–Owner Interaction subscale) with their service dogs during the COVID-19 lockdown compared to the general context prior to the COVID-19 lockdown. Interestingly, some factors modulated the effects of the COVID-19 lockdown on the recipient–service dog relationships, such as the service dog’s behavioral changes, the recipient’s stress level, and the decrease in the time taken for walks, as well as the dyad’s living environment.

Our results revealed that the COVID-19 lockdown enhanced Perceived Emotional Closeness between recipients and their service dogs. A similar effect was observed during the COVID-19 lockdown in Spanish populations and their pets [18]. Modulation of emotional closeness included notably the trauma the recipients pictured experiencing if their service dog died and the tendency to tell it things they would not tell anyone else. As previously reported, service dogs clearly appear as a confidant [37] and a source of support that helped the owners to cope with the challenging COVID-19 lockdown [10,11,12,13,14]. Interestingly, recipients that owned more than one dog (i.e., other than their service dog)—but not other animals—felt emotionally closer to their service dog. Such cumulative effects should be explored further.

Our study revealed that the COVID-19 lockdown involved a decrease in dog–owner interactions. Specifically, recipients of service dogs played less with their dogs, bought them fewer presents and took them less often to visit people during the COVID-19 lockdown. If the changes related to the latter two activities are probably a direct consequence of the lockdown conditions (i.e., shops closed, strict restriction of time spent outdoors), the decrease in time spent playing is surprising. One could argue that lockdown conditions offered more time to play with pets, as reported in a British survey [22] and the Dogs Trust’s COVID-19 Report [38]. However, the participants in the present study were individuals with several disabilities that may compromise their ability to play inside their home (e.g., difficulty playing in a small indoor space with a service dog if the recipient is in a wheelchair). This assumption would explain why the interaction score of recipients without an outdoor space decreased more. Conversely, service dogs may not be used to playing inside their home, where they are mostly in their working role helping their recipient. Lastly, some service dogs seemed to display boredom due to a lack of stimulation that could impact interactions with humans [39]. However, one aspect of the Dog–Owner Interaction that improved during the COVID-19 lockdown was that the recipients hugged and kissed their service dogs more often. This corroborates the fact that, although interactions were affected by the COVID-19 lockdown, attachment to their service dog did not seem to be impaired per se. Taken together, we may remain circumspect about this decrease in Dog–Owner Interactions as the main changes concerned two items directly affected by lockdown conditions. One may argue that a revised version of the MDORS should have been used, as by Bowen et al. [21], who excluded three items in their study (i.e., How often do you take your dog to visit people? How often do you take your dog out in the car? How traumatic do you think it will be for you when your dog dies?). However, we considered using the whole tool would yield more information about human–dog relationships.

The most surprising and troubling change relates to the increase in Perceived Costs during the COVID-19 lockdown. Specifically, the main issue faced by the recipients was the difficulty of looking after their service dog and the fact that their service dog made too much mess. This latter information was reinforced by the fact that recipients felt more costs in taking care of their service dogs when they displayed behavioral changes, especially boredom, excitement and frequent vocal manifestations. These behavioral changes were not specific to our study since other surveys, including pet dogs, reported similar modifications [15,18,22] that could be costly for people with disabilities. We propose two non-exclusive hypotheses to explain why such difficulties increased when the recipients felt more stressed. On the one hand, being more stressed may have increased the recipients’ sensitivity to their service dog’s behavioral problems (i.e., already present before the COVID-19 lockdown) and hence led them to perceive these later as challenging. On the other hand, the recipients’ stress may have consequences on their service dog that elicited the development of behavioral problems (e.g., not present before the COVID-19 lockdown).

Interestingly, previous surveys focusing on pet dog owners reported that the Perceived Costs items tended to remain the same or be reduced during lockdown [18,21]. We suspect these adults have fewer or no needs (or difficulties) than the recipients in our study. Thus, Bowen et al.’s [18] explanation that the support gained by pet owners from their pets increased when the owner’s quality of life was more impaired does not seem to apply to everyone. Our subjects who, in addition to their disabilities, reported more costs were those who felt more stressed during the COVID-19 lockdown, had no garden and took shorter walks. All these traits are elements that could lead to a reduction in their quality of life during the COVID-19 lockdown. Since people with disabilities are already in vulnerable situations (e.g., altered physical, health and mental conditions), the costs in terms of social isolation (e.g., no outing, no shopping, no visit to museums) and their anxiety would be exacerbated. Moreover, reports have shown that owning pets can intensify depressive symptoms in depressive individuals due to too many responsibilities [40]. Finally, we cannot exclude the fact that differences between ours and other studies [18,21] could be linked to the time between the beginning of the lockdown and the timing of the survey (i.e., 3 weeks versus 10 weeks in our study). The changes in costs perceived could have taken longer to emerge than other dimensions of the human–dog relationship measured by MDORS. Indeed, as Hinde [1] stated: “the progress of a relationship is often traced through periods of marked change or crisis, but it is essential to remember that change may also be gradual, occurring in the course of everyday life”. Longitudinal studies in crisis situations would be useful to further understand human–dog relationship dynamics.

The service dogs in our sample were reported to have displayed behavioral changes during the COVID-19 lockdown, as reported for pet dogs [15,18,22]. Our study found that approximately half of the service dogs displayed behavioral changes, thus agreeing with Bowen et al.’s [18] report but not other studies [15,22]. These discrepancies could be explained by methodological differences (e.g., standardized questionnaire, diary data or directed content analysis). Similar to Bowen et al. [18] and the Dogs Trust’s COVID-19 Report [38], more human attention-seeking was the most reported behavioral change, followed by vocal emissions (e.g., barking, whining). Boredom levels also commonly changed [15,22]. Holland et al. [22] reported that owners perceived a state of depression in their pet dogs as well. These authors also reported that owners explained this change in their pet dog by the reduced variety of activities, insufficient exercise and stimulation associated with the COVID-19 lockdown since we found a reduction of walking time. All the other previously mentioned studies also observed other behavioral changes. Thus, as lockdown clearly impacted human well-being (e.g., [10,11,12,13,14], this might also have been the case for both pet and service dogs. As Bowen et al. [18] mentioned, such behaviors need to be taken into consideration as they “could easily lead to other problems if the lockdown continued or these changes were mishandled by owners”.

The generalizability of our findings is limited by several factors. First, our sample size (*n* = 70) was smaller compared to other studies on similar topics (e.g., 5926 participants [17]; 4105 participants [15]; 1297 participants [18]). Nevertheless, our sample focused on service dog recipients, not on pet dog owners. Additionally, contrary to previous investigations based on convenience samples (i.e., mainly animal lovers), we used a list of recipients of service dogs, and this limited the eventual recruitment bias. Another limitation concerns our survey design. Due to the sudden COVID-19 lockdown, we could not apply a strict longitudinal design with a before and during lockdown data collection. We could only collect one set of data points from questionnaires asking respondents to provide answers for two specific periods (i.e., prior to and during lockdown), i.e., thus including a form of retrospective survey. However, since the information reported referred to a period that had occurred not long prior to the data collection, the quality of the report could be considered excellent [41]. Other limitations should be addressed since we had a limited ability to examine the relationships between the variables. In the present study, we did not perform direct observations of the relationships. Thus there is a lack of objective measures of the dogs’ behavior and welfare (self-reported by the handler). We also need to be cautious about the generalizability of these results since the sample was from one specific service dog provider. We only provided a broad explanation of what was intended by the “general context” situation. Repeating this information at each item of the MDORS could have better aided the recipients in their responses throughout the survey. Our sample was mainly composed of individuals with physical disabilities, and these results may be more specific to this population and not to all recipients of service dogs. Further studies should compare people with different psychological and/or physical disabilities.

## 5. Conclusions

To conclude, our study confirms the fact that service dogs constituted a source of emotional support for their owners during the COVID-19 lockdown, as previously shown with other pets. Service dogs fulfil the role of a pet as well as their professional role (e.g., perform a specific physical or functional task(s) to aid their owners). However, contrary to previous observations of pet dog owners, it appears that the COVID-19 lockdown elicited a costlier relationship for service dog recipients. Our study highlights the fact that, in extreme situations, characteristics of human–animal relationships can be exacerbated, both positively and negatively. These results are of importance for organizations delivering service dogs. For example, using the MDORS at key moments of the service dogs’ life would give organizations an objective tool to measure and follow the quality of their relationship with their recipient and evaluate the support the organizations might have to provide. For example, an increase in the Perceived Costs subscale could be a warning sign. In a crisis, they must be sufficiently aware of taking into account the recipients’ difficulties with their service dogs and their daily living issues since both factors could have negative impacts on the service dogs and their relationship with their owners. Some warning factors (e.g., living alone, having no outdoor space), as we highlighted here, are indicators of the need for closer monitoring and better assistance. As suggested by Bowen et al. [18], this type of research should be reproduced at the international level to better understand what happens between animals and owners in social deprivation contexts, and especially how human–animal relationships are affected by this context. Since relationships are dynamic over time and are influenced by factors that are extrinsic and intrinsic to each individual [1], it would provide a unique opportunity to better understand the dynamic nature of human–animals relationships.

## Figures and Tables

**Table 1:** Characteristics of our sample.

Information about Recipients		Information about Service Dogs	
Gender	47 ♀ 23 ♂	Mean age in years (range)	5.7 ± 2.5 (2–12)
Mean age in years (range)	37.4 ± 15.3 (8–72)	Mean time since attribution to recipient (months)	42.0 ± 30.7
Under 18 yo/Over 18 yo	10/60	Sex	33 ♀ 37 ♂
Activity (and change during lockdown)		Breed	
*Employed*	16 (15)	*Labradors*	35
*School/specialized institution*	11 (11)	*Golden retrievers*	34
Highest level of education (for recipient or for parents if recipient was under 18 yo)		*No information*	1
*High school diploma or less*	38	Dog type	
*Bachelor’s degree*	15	*For individuals with physical disabilities*	62
*Master’s degree*	11	*For ASD children*	7
*Above Master’s degree*	6	*For epileptic people*	1
Employment status (for recipient or parents if recipient was under 18 yo)		Changes in behavior during lockdown	
*Tradesman, company head*	1	*All behaviors (Yes/No)*	34/36
*Manager and intellectual worker*	10	*Boredom*	14/56
*Employee*	17	*Excitement*	5/65
*Student*	2	*Destruction of objects*	2/68
*Retired*	2	*Vocal noises*	11/59
*Unemployed*	38	*Attention seeking*	22/48
Condition of housing during lockdown		*Other*	18/52
*Alone or not*	20/50		
*Regular housing or not*	65/5		
*Flat, house or other*	25/45/0		
*Urban, semi-urban or rural area*	25/20/25		
*Presence of outside area or not*	61/9		
*Regional location (green/red zones)*	46/24		

yo—years old.

**Table 2:** Scores for items on each subscale of the MDORS during the COVID-19 lockdown and in a general context (mean ± SD). Significant differences between contexts (i.e., the COVID-19 lockdown vs. General context prior to COVID-19 lockdown) are in bold—level of significance: *p* < 0.05 (Friedman tests). Number of item corresponds to the order of items in the MDORS questionnaire developed by Dwyer et al. [19].

	Perceived Emotional Closeness Subscale			Perceived Costs Subscale			Dog–Owner Interaction Subscale		
	General Context	COVID-19 Context	Wilcoxon Test	General Context	COVID-19 Context	Wilcoxon Test	General Context	COVID-19 Context	Wilcoxon Test
Item 1	4.53 ± 0.76	4.53 ± 0.83	Z = 0.03, *p* > 0.05	1.37 ± 0.82	1.34 ± 0.80	Z = 0.43, *p* > 0.05	4.13 ± 1.30	4.26 ± 1.28	**Z = 2.52,** ***p* = 0.012**
Item 2	4.56 ± 0.79	4.59 ± 0.75	Z = 0.46, *p* > 0.05	1.64 ± 1.18	1.63 ± 1.23	Z = 0.68, *p* > 0.05	3.93 ± 0.79	4.23 ± 0.78	**Z = 3.24,** ***p* = 0.001**
Item 3	4.84 ± 0.58	4.87 ± 0.56	Z = 0.80, *p* > 0.05	1.37 ± 1.01	1.44 ± 0.99	Z = 0.20, *p* > 0.05	4.29 ± 0.93	2.87 ± 1.58	**Z = 5.11,** ***p* < 0.001**
Item 4	4.69 ± 0.75	4.70 ± 0.69	Z = 0.28, *p* > 0.05	1.87 ± 1.15	2.20 ± 1.37	**Z = 2.76,** ***p* = 0.006**	3.29 ± 1.02	2.76 ± 1.26	**Z = 3.76,** ***p* < 0.001**
Item 5	4.61 ± 0.75	4.77 ± 0.66	Z = 1.70, *p* > 0.05	1.64 ± 1.19	1.63 ± 1.07	Z = 0.96, *p* > 0.05	3.70 ± 0.92	3.60 ± 1.12	Z = 1.07, *p* > 0.05
Item 6	4.46 ± 0.76	4.50 ± 0.74	Z = 0.60, *p* > 0.05	1.56 ± 0.96	1.64 ± 1.05	Z = 0.38, *p* > 0.05	3.47 ± 1.02	3.56 ± 1.04	Z = 1.30, *p* > 0.05
Item 7	4.26 ± 1.09	4.27 ± 1.13	Z = 0.84, *p* > 0.05	1.33 ± 0.83	1.37 ± 0.97	Z = 0.53, *p* > 0.05	4.50 ± 0.74	4.70 ± 0.62	**Z = 3.18,** ***p* = 0.002**
Item 8	4.57 ± 0.79	4.64 ± 0.82	Z = 1.27, *p* > 0.05	1.73 ± 1.15	1.69 ± 1.10	Z = 0.74, *p* > 0.05	4.61 ± 0.62	4.66 ± 0.59	Z = 1.10, *p* > 0.05
Item 9	4.66 ± 0.70	4.74 ± 0.65	**Z = 2.02,** ***p* = 0.043**	1.44 ± 0.93	1.66 ± 1.10	**Z = 2.92,** ***p* = 0.004**	-	-	-
Item 10	3.41 ± 1.25	3.61 ± 1.30	**Z = 2.82,** ***p* = 0.005**	-	-	-	-	-	-
Total score	44.59 ± 5.21	45.23 ± 4.9	**Z = 1.99,** ***p* = 0.047**	13.96 ± 5.88	14.6 ± 6.05	**Z = 2.17,** ***p* = 0.030**	31.91 ± 4.47	30.63 ± 4.54	**Z = 3.30,** ***p* < 0.001**
Mean score	4.46 ± 0.52	4.52 ± 0.49	**Z = 2.07,** ***p* = 0.038**	1.55 ± 0.65	1.62 ± 0.67	**Z = 2.10,** ***p* = 0.036**	3.99 ± 0.56	3.83 ± 0.57	**Z = 3.30,** ***p* < 0.001**

**Perceived Emotional Closeness subscale**: Item 1: my dog helps me get through tough times; Item 2: my dog is there whenever I need to be comforted; Item 3: if everyone else left me, my dog would still be there for me; Item 4: I would like to have my dog near me all the time; Item 5: my dog provides me with constant companionship; Item 6: my dog is constantly attentive to me; Item 7: My dog gives me a reason to get up in the morning; Item 8: I wish my dog and I never had to be apart; Item 9: How traumatic do you think it will be for you when your dog dies? Item 10: How often do you tell your dog things you don’t tell anyone else? **Perceived Costs subscale**: Item 1: How often do you feel that looking after your dog is a chore? Item2: How often does your dog stop you doing things you want to? Item 3: How often do you feel that having a dog is more trouble than it is worth? Item 4: How hard is to look after your dog? Item 5: There are major aspects of owning a dog I don’t like; Item 6: It is annoying that I sometimes have to change my plans because of my dog; Item 7: It bothers me that my dog stops me doing things I enjoyed doing before I owned it; Item 8: My dog costs too much money; Item 9: My dog makes too much mess; **Dog–Owner Interaction subscale**: Item 1: How often do you kiss your dog? Item 2: How often do you play games with your dog? Item 3: How often do you take your dog to visit people? Item 4: How often do you buy your dog presents? Item 5: How often do you give your dog food treats? Item 6: How often do you groom your dog? Item 7: How often do you hug your dog? Item 8: How often do you have your dog with you while relaxing, i.e., watching TV?

## Data Availability

The data presented in this study are available on request from the corresponding author.
